# Readiness for competition across sports and genders: a study on psychological skills intervention

**DOI:** 10.3389/fpsyg.2025.1701631

**Published:** 2025-11-14

**Authors:** Stefan Alecu, Gheorghe Adrian Onea

**Affiliations:** Department of Physical Education and Special Motricity, Transilvania University, Brașov, Romania

**Keywords:** sport motivation, readiness for competition, mental skills training, competitive anxiety, psychological intervention

## Abstract

**Introduction:**

Psychological readiness is essential for athletic performance, particularly under competitive pressure. While physical training remains critical, growing evidence supports the role of psychological interventions in enhancing motivation and managing pre-competition anxiety. This study, grounded in Self-Determination Theory (SDT), evaluates the effects of a structured 4-week psychological program on athletes’ motivation and anxiety levels.

**Materials and methods:**

A total of 512 athletes (210 individual-sport, 302 team-sport; 280 women, 232 men) completed the Sport Motivation Scale-II (SMS-II) and the Competitive State Anxiety Inventory-2 (CSAI-2) before and after a 4-week intervention. The program included goal-setting, visualization, mindfulness, and team cohesion sessions, delivered by certified sport psychologists and applied by coaches. Data were analyzed using mixed ANOVAs, t-tests, Bonferroni correction, and effect size estimates (Cohen’s *d*, *η*^2^).

**Results:**

Post-intervention results showed substantial improvement in intrinsic, integrated, and identified motivation, alongside decreases in introjected and external regulation and amotivation (*p* < 0.001). Athletes also experienced significant reductions in cognitive and somatic anxiety and increased self-confidence. These effects were large (*d* > 0.8) and consistent across gender and sport types; however, no interaction effects remained reliable after correction.

**Conclusion:**

A brief psychological intervention can significantly improve motivational regulation and competitive readiness across diverse athlete groups. These findings highlight the value of integrating mental skills training into routine sport preparation and support the implementation of scalable, evidence-based psychological programs.

## 1 Introduction

In competitive sports, psychological readiness is increasingly recognized as a key determinant of athletic performance, particularly under pressure-laden conditions. Beyond physical preparation, athletes are expected to regulate emotions, maintain motivation, and manage performance-related anxiety in high-stakes environments. While physical training has traditionally dominated sport development programs, a growing body of literature emphasizes the importance of structured psychological support in optimizing not only well-being but also performance outcomes (Reinebo et al., 2024; Williamson et al., 2022).

One widely accepted theoretical framework for understanding athlete motivation is Self-Determination Theory (SDT) (García Calvo et al., 2010; Torre, 2024; Howard et al., 2021). SDT distinguishes between self-determined (autonomous) motivation, such as intrinsic motivation, integrated regulation, and identified regulation, and less self-determined forms, including introjected and external regulation, as well as amotivation. Research in sport settings has shown that athletes who are more autonomously motivated tend to experience higher levels of persistence, satisfaction, emotional resilience, and performance efficacy (Levine et al., 2022; Warmath et al., 2021). Interventions that support psychological needs—autonomy, competence, and relatedness—have been shown to enhance self-determined motivation across various levels of sport and disciplines. While other theoretical perspectives, such as Achievement Goal Theory (Nicholls, 1984; Elliot and Dweck, 2005) and constructs like Mental Toughness (Crust and Clough, 2011) or Resilience frameworks (Fletcher and Sarkar, 2013) have also been widely applied to explain athletes’ psychological functioning, Self-Determination Theory (SDT) provides a particularly comprehensive basis for the present study. SDT not only accounts for the quality of motivation but also offers a direct link between psychological needs (autonomy, competence, and relatedness) and well-being outcomes, making it highly suitable for interventions targeting both performance and emotional regulation. In contrast, goal-oriented models primarily emphasize achievement striving, while trait-based frameworks, such as mental toughness, focus on dispositional characteristics rather than change mechanisms. Given our study’s emphasis on modifiable motivational processes through psychological skills training, SDT was considered the most relevant framework to guide both intervention design and outcome assessment.

Alongside motivation, another critical construct influencing athletic performance is competitive state anxiety (Chun et al., 2022; Tsopani et al., 2011; Shi et al., 2024; Patel et al., 2010; Mullen and Jones, 2021). According to multidimensional anxiety theory and the revised Competitive State Anxiety Inventory-2 (CSAI-2), pre-competition anxiety can be separated into cognitive anxiety (e.g., worry, negative thoughts), somatic anxiety (e.g., physiological arousal), and self-confidence (Martens et al., 1990). High levels of anxiety, particularly cognitive anxiety, are consistently linked to performance decrements, especially when not accompanied by high self-confidence (Craft et al., 2003; Grossbard et al., 2009). Conversely, self-confidence has been identified as a protective factor that buffers the negative effects of pre-competitive anxiety and contributes to effective coping, focus, and goal execution.

Despite considerable progress in sport psychology, several critical gaps persist in the literature that our study seeks to address. Firstly, while traditional psychological skills training (PST) interventions (Lauria et al., 2016; Asken et al., 2021)—such as goal-setting and imagery—have shown robust effects on reducing state anxiety, there remains notable heterogeneity in outcomes across sport types and individual characteristics (e.g., age, gender, level). Meta-analyses suggest that more nuanced research is needed to explore how intervention effectiveness varies between individual and team sports, as well as across genders, given that these moderating factors remain underexplored (Li et al., 2025).

Secondly, although PST has demonstrated large effects in reducing competitive anxiety, its impact on motivation profiles, particularly through Self-Determination Theory constructs such as integrated regulation and amotivation, has received limited empirical attention (Alkasasbeh and Akroush, 2025). Additionally, the majority of intervention studies do not simultaneously assess both motivational dynamics and anxiety outcomes, which limits a comprehensive understanding of psychological readiness for competition.

Third, intervention research in sport psychology largely focuses on performance optimization in healthy athletes, with a dearth of studies on athletes with clinical or subclinical mental health issues (e.g., anxiety disorders, depressive symptoms) (Ekelund et al., 2022). Moreover, the research-practice gap remains substantial: practitioners often lack accessible, evidence-based materials grounded in research, and researchers seldom integrate practical feasibility or ecological context in their interventions (Keegan et al., 2017).

Also, given the well-established role of psychological factors in athletic performance, many training programs lack systematic psychological intervention strategies (Reinebo et al., 2024; di Fronso and Budnik-Przybylska, 2023; Wang et al., 2025) that can be implemented by coaches in real-world settings. Furthermore, while numerous studies have examined psychological variables independently, few have investigated how short-term, structured psychological interventions can simultaneously influence both motivation and anxiety-related readiness, particularly in large and diverse athlete samples. Additionally, evidence remains mixed regarding how individual versus team sport athletes, as well as male vs. female athletes, respond to such interventions, leaving important questions about generalizability and personalization unaddressed.

To address these gaps, the present study implemented a 4-week psychological intervention targeting goal setting, visualization, mindfulness, and team cohesion. It evaluated its impact on athletes’ motivational regulation (via SMS-II) and competitive readiness (via CSAI-2). The intervention was delivered by certified sport psychologists and implemented by coaches across both individual and team sports. With a large sample of 512 participants and a pre-post design, the study aimed to assess changes in motivation and anxiety while also examining whether sport type and gender moderated these effects.

While psychological interventions have been widely studied in Western and Anglophone sport contexts, research on athletes from Eastern European countries, including Romania, remains limited. Romanian athletes often train within highly structured, coach-centered systems that emphasize discipline and external evaluation, conditions that may increase vulnerability to controlled forms of motivation and competitive anxiety. Moreover, access to systematic psychological support remains uneven across Romanian sport institutions. By examining the effects of a structured intervention in this underrepresented context, the present study contributes to a more globally inclusive understanding of how Self-Determination Theory and mental skills training can enhance motivation and emotional regulation. This cultural lens not only enhances the generalizability of our findings but also provides practical insights for sport psychologists and coaches working in similar performance settings.

### 1.1 Objectives and hypotheses

The primary objective of this study was to evaluate the effects of a four-week psychological skills training (PST) intervention on athletes’ motivation regulation and competitive anxiety/self-confidence. We aimed to assess changes in both motivational quality and psychological readiness for competition before and after the intervention.

A secondary objective was to examine whether the intervention’s impact differed by sport type (individual vs. team sports) and gender, given previous evidence suggesting that these factors may moderate psychological responses to competitive stress and intervention outcomes.

*H1*: Athletes will show a significant increase in self-determined forms of motivation (intrinsic, integrated, and identified regulation) and a decrease in controlled forms (introjected and external regulation) and amotivation following the intervention.

*H2*: Athletes will report a significant decrease in cognitive and somatic anxiety and a significant increase in self-confidence from pre- to post-intervention.

*H3*: The intervention will yield greater improvements in psychological readiness in individual-sport athletes compared to team-sport athletes.

*H4*: Female athletes will show a significantly greater increase in intrinsic motivation and self-confidence and a greater anxiety reduction compared to male athletes.

## 2 Materials and methods

### 2.1 Design of the research

This study employed a quasi-experimental, within-subjects, pre-post intervention design to evaluate the effectiveness of a structured psychological skills training (PST) program (Weinberg and Williams, 2015) on motivational regulation and competitive anxiety in athletes from both individual and team sports (Zakrajsek and Blanton, 2017). The research design was selected in accordance with ethical and logistical constraints related to intervention-based research in elite sport contexts, where randomization and control groups are not always feasible.

The study was conducted between April 2023 and May 2025. Data were collected at two distinct time points:

T1 – Pre-Intervention. All participants completed two psychometrically validated self-report instruments: the Sport Motivation Scale II (SMS-II) and the Competitive State Anxiety Inventory-2 (CSAI-2). Assessments were administered during the final 5–7 days prior to a major competition (national championship, international competition, or elite-level tournament), during a standardized rest period to control for acute training-related fluctuations. No psychological intervention was delivered prior to this assessment.

T2 – Post-Intervention. Four weeks prior to a subsequent high-stakes competition of comparable importance and structure, the same cohort of participants engaged in a four-week psychological skills training intervention. Following the completion of the intervention and within 48–72 h prior to the target competition, athletes again completed the SMS-II and CSAI-2 instruments under identical conditions to those used at T1.

To minimize intra-individual variability, the post-intervention assessments were timed to match the competitive context, time of day, and physical state of the pre-intervention measures. No participants reported any injuries, illnesses, or training modifications during the 4-week intervention that would compromise their performance or self-report accuracy.

Due to ethical and scheduling constraints in competitive environments, a control group was not feasible; therefore, causal inferences should be interpreted with caution.

### 2.2 Participants

A total of 512 competitive athletes (*M*_age = 20.8 years, SD = 2.7; range = 16–26 years) participated in this study. The sample consisted of 280 female athletes (54.7%) and 232 male athletes (45.3%), drawn from sports clubs nationwide, national training centers, and elite sport development programs. All participants were competitive Romanian athletes affiliated with national training centers, regional clubs, or elite development programs across Romania and had a minimum of 3 years of competitive experience in their respective sports. They were actively preparing for national or international competitions at the time of data collection.

Participants were stratified into individual sports (*n* = 210; 41.0%) and team sports (*n* = 302; 59.0%) as follows: Individual sports (*n* = 210): *Skiing*: 42 women (*M*_age = 20.4 ± 2.6), 46 men (*M*_age = 20.9 ± 2.5); total = 88; *Athletics*: 68 women (*M*_age = 19.8 ± 2.9), 54 men (*M*_age = 21.1 ± 2.3); total = 122. Team Sports (*n* = 302): *Handball*: 82 women (*M*_age = 21.2 ± 2.4), 64 men (*M*_age = 21.6 ± 2.1); total = 146, *Volleyball*: 90 women (*M*_age = 20.1 ± 2.7), 66 men (*M*_age = 20.5 ± 2.5); total = 156. The distribution of participants across sport types and genders was approximately balanced, with a slightly higher representation from team sports. Age distributions were approximately normal across all subgroups, and no outliers were detected.

Eligible participants were athletes aged 16–26 years who were actively competing in individual (skiing, athletics) or team sports (handball, volleyball) at a national or regional level, with a minimum of 3 years of competitive experience. All athletes aged 18 and above provided informed consent. For minors (ages 16–17), parental or guardian consent and participant assent were obtained in compliance with institutional and ethical guidelines.

Inclusion required participation in a major competition during the study period, completion of both SMS-II and CSAI-2 assessments at pre- and post-intervention (T1 and T2), and full attendance in the 4-week psychological intervention.

The exclusion criteria included injury, illness, or absence affecting study participation, missing more than one intervention session, incomplete questionnaire data, or involvement in other psychological training programs during the study. Athletes with known psychiatric or neurological conditions or those competing at non-elite or recreational levels were also excluded.

**Figure 1:**
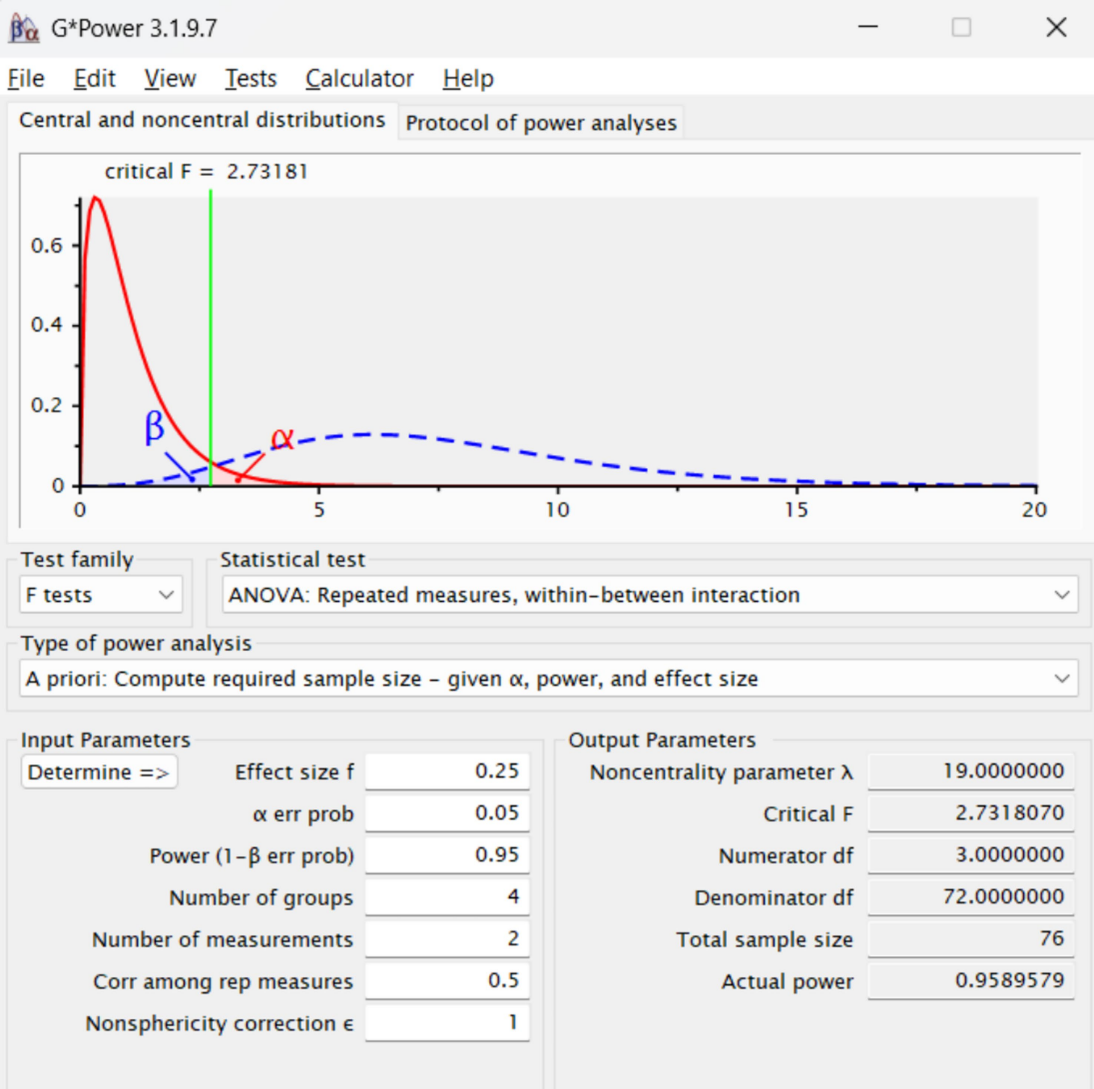
Statistical power analysis.

All participants (or guardians) provided written informed consent in accordance with the Declaration of Helsinki and institutional guidelines for research involving human subjects (Figure 1).

An *a priori* power analysis was conducted using G*Power 3.1.9.7 to determine the minimum required sample size for detecting within-between interaction effects in a mixed-design ANOVA. The analysis assumed a medium effect size (*f* = 0.25), a conventional alpha level of 0.05, and a desired statistical power of 0.95. The design included four independent groups (2 genders × 2 sport types), two repeated measures (pre- and post-intervention), an assumed correlation among repeated measures of 0.50, and no violation of sphericity (*ε* = 1).

The results indicated that a minimum sample size of *N* = 76 would be required to detect an interaction effect with sufficient statistical power. Given that the actual study included 512 participants, the obtained power was substantially higher (1.000), confirming that the study was statistically robust for detecting medium-sized effects or larger.

### 2.3 Intervention framework

The psychological intervention followed a standardized, evidence-based sport psychology curriculum developed in accordance with best practices from applied sport psychology literature (Bordo et al., 2025; Lange-Smith et al., 2023).

Psychological Skills Training (PST) is a structured, multimodal intervention that combines mental strategies, including goal setting, imagery, self-talk, relaxation, and mindfulness. Its purpose is to enhance performance, emotional control, and self-regulation in athletes. Research strongly supports the efficacy of PST in improving both psychological skills and sports outcomes (Zakrajsek and Blanton, 2017). The model framework, from Vealey (1988) and widely used in applied sport settings, PST follows three progressive phases: (1) Education phase – Athletes learn about the purpose and benefits of mental skills (e.g., “what is goal-setting? and why does visualization work?”); (2) Acquisition phase – Practical instruction and practice of skills such as deep breathing, imagery scripts, or goal formulation; (3) Practice phase – Skills are integrated into physical practice and competitive scenarios to promote automaticity and transfer of learning. This tripartite model supports gradual skill development and has strong empirical grounding.

The intervention was supervised by a licensed sport psychologist certified by the Romanian College of Psychologists and delivered by each team’s head coach, who was trained and provided with standardized manuals, scripts, and fidelity checklists to ensure intervention consistency across contexts.

Presented in Table 1, each week of the intervention targeted a distinct psychological skill, with sessions embedded into regular team practice routines or conducted immediately following training to maximize ecological validity: Week 1: Goal-setting implementation of team-based and individual goal-setting frameworks (SMART model), including athlete-driven performance, process, and outcome goals. Week 2: Visualization and mental rehearsal guided imagery sessions focused on performance optimization, scenario rehearsal, and emotional regulation in competition-like environments. Week 3: Breathing and mindfulness training instruction and practice of physiological self-regulation techniques, including box breathing, body scan meditations, and attention refocusing exercises to reduce pre-competition somatic arousal. Week 4: Team cohesion and reflective practices facilitation of trust-building exercises, shared storytelling, and post-practice group reflections to strengthen psychological safety, inter-athlete support, and collective efficacy.

**Table 1:** A 4-week sport psychology intervention program.

Week	Theme	Exercise	Description	Format	Duration/repetitions
1	Goal-setting	Team goal-setting meeting	Players and coaches define shared goals (performance, behavior, mindset).	Group workshop	45–60 min
		SMART individual goals	Each player writes 1–2 SMART goals with coach feedback.	Solo and coach review	20 min writing and 10 min feedback/player
		Goal visualization	Visualization of achieving personal and team goals.	Guided imagery session	10 min per session × 2 sessions
2	Visualization and mental rehearsal	Performance imagery	Mental rehearsal of key sport-specific actions (shooting, passing).	Guided team session	10–12 min × 3 sessions
		Highlight reel playback	Players recall and visualize 2–3 past personal highlights to boost confidence.	Individual reflection	5 min before each practice
		Coping imagery	Visualization of calmly handling setbacks (missed shots, pressure).	Guided imagery	15 min × 1 session
3	Breathing and mindfulness	Box breathing	Structured breathing: inhale, hold, exhale, hold – all for 4 s.	Coach-led breathing	5–8 min before each session
		Body scan meditation	Athletes bring attention to different body parts to promote relaxation and awareness.	Audio or coach-led	10 min × 2 sessions
		Reset routine drill	Players practice pre-action routine (deep breath and cue word before key moments).	On-court/practice drill	5 reps/player per session
4	Team cohesion and reflection	Trust circle	Players share one personal goal and one fear to build openness and trust within the team.	Group session	30–40 min × 1 session
		Team-building challenge	Cooperative physical or communication-based challenge (obstacle course, blindfold maze).	Physical activity	45 min × 1 session
		Post-game reflection	Players reflect together after matches: 1 positive, 1 improvement, and team support.	Guided debrief	10–15 min after each game

Coaches submitted weekly fidelity reports, which included session duration, athlete attendance, adherence to protocol, and subjective assessments of athlete engagement. The supervising sport psychologist reviewed fidelity logs weekly and provided brief follow-ups to reinforce protocol alignment. Athletes were not informed that psychological outcomes were the focus of the study to reduce response expectancy effects.

### 2.4 Instruments of assessment

To comprehensively assess the psychological state of athletes preparing for high-stakes competition, we selected the Sport Motivation Scale II (SMS-II) and the Competitive State Anxiety Inventory-2 (CSAI-2) due to their strong theoretical foundations and validated use in sport psychology research. The SMS-II, grounded in Self-Determination Theory (Flannery, 2017; Huang et al., 2022; Lourenço et al., 2022), measures distinct forms of motivation ranging from intrinsic to amotivation, providing insight into the quality of athletes’ motivational regulation. The CSAI-2 assesses pre-competitive anxiety and self-confidence, measuring the emotional and physiological responses that directly impact performance. Together, these two instruments offer a multidimensional view of athletes’ psychological readiness for competition, aligning closely with the aims of this intervention-based study. Although the SMS-II and CSAI-2 scales were both used to assess psychological dimensions relevant to competitive performance, they differ substantially in structure and scoring ranges. The SMS-II subscales consist of 3 items each (range: 3–21), while the CSAI-2 (Lundqvist and Hassmén, 2005; Coelho et al., 2010) has a similar structure. Internal consistency for each subscale was assessed using Cronbach’s alpha (*α*) based on the present sample. For the SMS-II, α values ranged from 0.78 to 0.87 across subscales, indicating acceptable to good reliability. For the CSAI-2 subscales, internal consistency was similarly strong, with *α* = 0.84 for Cognitive Anxiety, *α* = 0.81 for Somatic Anxiety, and *α* = 0.89 for Self-Confidence. These values support the reliability of the instruments in this Romanian athlete sample.

The subscales include nine items (range: 9–63). As such, raw scores cannot be directly combined or entered into multivariate models (e.g., MANOVA or regression) without introducing artificial weighting due to scale length rather than psychological significance. To address this, all subscale scores were first converted into standardized z-scores, allowing for meaningful comparison and integration across instruments. This standardization enabled the creation of a composite Psychological Readiness Index (PRI), which represents a balanced profile of athletes’ motivation and emotional readiness for competition.

To assess the athletes’ global psychological readiness for competition, we developed a composite Psychological Readiness Index (PRI) by integrating standardized scores (*z*-scores) from selected subscales of the SMS-II (Rodrigues et al., 2021; Chin et al., 2021) and CSAI-2 instruments. The index captures the balance between positive psychological resources (autonomous motivation and self-confidence) and negative performance-related states (controlled motivation and competitive anxiety). Positive contributors to the index include intrinsic motivation, integrated regulation, identified regulation, and self-confidence. Negative contributors include introjected regulation, external regulation, amotivation, cognitive anxiety, and somatic anxiety.

The Psychological Readiness Index (PRI) was constructed as a composite, exploratory metric integrating key dimensions from the SMS-II (motivation) and CSAI-2 (anxiety/confidence) to provide an overall profile of psychological readiness. All subscale scores were standardized (z-scores) to account for differences in scale lengths and variances, and then combined using equal weighting. While the PRI has not been independently validated as a psychometric construct, its design was grounded in self-determination theory and competitive anxiety frameworks. Specifically, positively valenced indicators (intrinsic motivation, self-confidence) were summed, and negatively valenced ones (amotivation, cognitive anxiety) were subtracted to yield a single composite score. This approach follows conventions used in previous exploratory indices and was intended to offer a high-level, descriptive overview of athletes’ psychological states rather than serve as a validated diagnostic tool. Results based on the PRI should therefore be interpreted cautiously and seen as supplementary to the main subscale analyses.

All subscale scores were first converted into *z*-scores to account for differing scale lengths and variances. The PRI was then calculated by summing the positive *z*-scores and subtracting the sum of the negative *z*-scores, as per the formula:

PRI = (*Z*_Intrinsic_ + *Z*_Integrated_ + *Z*_Identified_ + *Z*_SelfConfidence_) − (*Z*_Introjected_ + *Z*_External_ + *Z*_Amotivation_ + *Z*_CognitiveAnxiety_ + *Z*_SomaticAnxiety_).

Higher PRI values indicate greater psychological readiness for competition, characterized by high self-determined motivation and confidence with low anxiety and controlled/amotivation. Lower PRI values reflect reduced readiness, typically associated with performance apprehension and lack of internalized motivation.

Table 2 provides an overview of the components included in the Psychological Readiness Index (PRI). The index integrates key motivational and emotional factors derived from the SMS-II and CSAI-2 instruments. Subscales associated with autonomous motivation and self-confidence contribute positively to readiness, while those reflecting controlled motivation, amotivation, and competitive anxiety contribute negatively. Each component is categorized by its source, psychological type, and directional influence on overall psychological readiness for competition.

**Table 2:** Overview of the components included in the Psychological Readiness Index (PRI).

Component	Source	Type	Contribution to PRI
Intrinsic motivation	SMS-II	Autonomous	Positive (↑ readiness)
Integrated regulation	SMS-II	Autonomous	Positive (↑ readiness)
Identified regulation	SMS-II	Controlled/self-endorsed	Positive
Self-confidence	CSAI-2	Emotional strength	Positive (↑ readiness)
Introjected regulation	SMS-II	Ego/Guilt-based	Negative (↓ readiness)
External regulation	SMS-II	Extrinsic	Negative
Amotivation	SMS-II	Lack of motivation	Negative
Cognitive anxiety	CSAI-2	Worry	Negative (↓ readiness)
Somatic anxiety	CSAI-2	Physical symptoms	Negative

### 2.5 Statistical analysis

All statistical analyses were conducted using SPSS version 26.0 and G*Power 3.1.9.7. Descriptive statistics, including means, standard deviations, coefficients of variation, 95% confidence intervals, skewness, and kurtosis, were computed for all subscales of the Sport Motivation Scale-II (SMS-II) and the Competitive State Anxiety Inventory-2 (CSAI-2) at both time points (T1 and T2). The Shapiro–Wilk test was applied to assess normality assumptions. Pre–post comparisons were conducted using paired sample *t*-tests to evaluate changes over time, and Cohen’s d effect sizes were calculated alongside percentage change from baseline. To assess the effects of the intervention across time and between groups, a mixed-design repeated-measures ANOVA (2 × 2 × 2) was performed for each subscale, with Time as a within-subjects factor and Gender and Sport Type as between-subjects factors. Partial eta squared (*η*^2^) was reported to quantify effect sizes. Partial eta squared (*η*^2^) was used to measure the magnitude of intervention effects, with values of 0.01, 0.06, and 0.14 typically interpreted as small, medium, and large effects, respectively (Cohen, 1988). Bonferroni correction was applied to control for Type I error due to multiple comparisons, setting the corrected alpha threshold at *p* < 0.0056. In addition, a MANOVA was conducted on the CSAI-2 subscales to assess multivariate effects across Cognitive Anxiety, Somatic Anxiety, and Self-Confidence. *Post hoc* power analysis using G*Power indicated that the achieved power was 1.000, confirming that the sample size of N = 512 was more than sufficient to detect medium-sized effects. An *a priori* power analysis confirmed that a minimum of 76 participants would be needed to achieve a power of 0.95, validating the adequacy of the study’s sample size.

## 3 Results

The descriptive statistics across the six SMS-II subscales and three CSAI-2 subscales are summarized in Tables 3, 4. These include mean scores, standard deviations, coefficients of variation (CV), 95% confidence intervals (CI), skewness, and kurtosis for both pre-intervention (T1) and post-intervention (T2) time points.

**Table 3:** Descriptive statistics for SMS-II subscales.

Sport type	Gender	Subscale	Time	*X*	SD	CV	95% CI		Skewness	Kurtosis	*p*
							Lower	Upper			
Individual sports	F	IM	T1	17.7	0.6	0.034	17.3	18.1	−0.12	−0.30	
			T2	19.2	0.5	0.026	18.8	19.6	−0.05	−0.35	0.003*
		INR	T1	17.1	0.7	0.041	16.6	17.6	−0.08	−0.25	
			T2	18.3	0.6	0.033	17.9	18.7	−0.03	−0.32	0.005*
		IDR	T1	17.5	0.6	0.034	17.1	17.9	−0.10	−0.28	
			T2	18.9	0.5	0.026	18.5	19.3	−0.04	−0.33	0.004*
		INTJ	T1	12.6	0.8	0.063	12.0	13.2	0.10	0.12	
			T2	11.1	0.7	0.063	10.6	11.6	0.08	0.10	0.030*
		EXT	T1	10.2	0.7	0.069	9.7	10.7	0.15	0.22	
			T2	9.3	0.6	0.065	8.9	9.7	0.10	0.18	0.022*
		AMOT	T1	6.3	0.9	0.143	5.7	6.9	0.30	0.50	
			T2	4.5	0.6	0.133	4.1	4.9	0.20	0.40	0.010*
	M	IM	T1	16.8	0.7	0.042	16.3	17.3	−0.14	−0.28	
			T2	18.6	0.6	0.032	18.1	19.1	−0.07	−0.31	0.005*
		INR	T1	16.4	0.8	0.049	15.8	17.0	−0.11	−0.22	
			T2	17.7	0.7	0.040	17.2	18.2	−0.06	−0.29	0.012*
		IDR	T1	17.0	0.7	0.041	16.5	17.5	−0.09	−0.24	
			T2	18.4	0.6	0.033	17.9	18.9	−0.04	−0.26	0.009*
		INTJ	T1	13.3	0.9	0.068	12.6	14.0	0.12	0.15	
			T2	11.7	0.8	0.068	11.1	12.3	0.09	0.14	0.038*
		EXT	T1	10.8	0.8	0.074	10.1	11.5	0.18	0.26	
			T2	9.9	0.7	0.071	9.4	10.4	0.14	0.22	0.031*
		AMOT	T1	7.1	1.0	0.141	6.4	7.8	0.34	0.55	
			T2	5.2	0.8	0.154	4.6	5.8	0.22	0.42	0.017*
Team sports	F	IM	T1	16.2	0.8	0.049	15.6	16.8	−0.25	−0.20	
			T2	18.0	0.7	0.039	17.4	18.6	−0.15	−0.32	0.004*
		INR	T1	15.8	0.8	0.051	15.2	16.4	−0.20	−0.18	
			T2	17.4	0.7	0.040	16.8	18.0	−0.11	−0.25	0.007*
		IDR	T1	16.4	0.7	0.043	15.9	16.9	−0.18	−0.20	
			T2	18.3	0.6	0.033	17.8	18.8	−0.12	−0.24	0.008*
		INTJ	T1	14.1	0.9	0.064	13.4	14.8	0.10	0.18	
			T2	12.3	0.9	0.073	11.6	13.0	0.06	0.12	0.035*
		EXT	T1	11.4	0.8	0.070	10.8	12.0	0.17	0.21	
			T2	10.2	0.7	0.069	9.7	10.7	0.12	0.20	0.026*
		AMOT	T1	7.5	1.0	0.133	6.8	8.2	0.29	0.47	
			T2	6.1	0.8	0.131	5.5	6.7	0.20	0.40	0.012*
	M	IM	T1	15.6	0.9	0.058	14.9	16.3	−0.28	−0.15	
			T2	17.4	0.8	0.046	16.8	18.0	−0.17	−0.21	0.006*
		INR	T1	15.1	0.9	0.060	14.4	15.8	−0.23	−0.18	
			T2	17.1	0.8	0.047	16.5	17.7	−0.13	−0.20	0.009*
		IDR	T1	15.9	0.8	0.050	15.3	16.5	−0.19	−0.17	
			T2	17.9	0.7	0.039	17.3	18.5	−0.12	−0.22	0.011*
		INTJ	T1	14.7	0.9	0.061	14.0	15.4	0.11	0.13	
			T2	12.9	0.8	0.062	12.3	13.5	0.08	0.12	0.042*
		EXT	T1	12.0	0.8	0.067	11.4	12.6	0.21	0.24	
			T2	10.8	0.7	0.065	10.3	11.3	0.16	0.20	0.030*
		AMOT	T1	8.2	1.0	0.122	7.5	8.9	0.30	0.45	
			T2	6.8	0.9	0.132	6.2	7.4	0.23	0.41	0.015*

X, mean; SD, standard deviation; CV, coefficient of variation; CI95%, confidence interval; p, significance threshold at *p* < 0.05 marked with *; IM, intrinsic motivation; INR, integrated regulation; IDR, identified regulation; INTJ, introjected; EXT, external; AMOT, amotivation.

**Table 4:** Descriptive statistics for CSAI-2 subscales.

Sport type	Gender	Subscale	Time	*X*	SD	CV	95% CI		Skewness	Kurtosis	*p*
							Lower	Upper			
Individual sports	F	CA	T1	28.8	0.9	0.031	28.2	29.4	0.10	−0.15	
			T2	24.3	1.0	0.041	23.7	24.9	0.05	−0.20	0.001*
		SA	T1	29.4	1.0	0.034	28.8	30.0	0.12	−0.10	
			T2	27.6	1.1	0.040	27.0	28.2	0.08	−0.18	0.002*
		SC	T1	50.8	1.2	0.024	50.2	51.4	−0.10	−0.22	
			T2	56.7	1.0	0.018	56.1	57.3	−0.12	−0.20	0.003*
	M	CA	T1	31.5	1.1	0.035	30.8	32.2	0.15	−0.10	
			T2	26.1	1.2	0.046	25.3	26.9	0.10	−0.12	0.002*
		SA	T1	32.1	1.1	0.034	31.5	32.7	0.18	−0.08	
			T2	29.7	1.2	0.040	29.1	30.3	0.14	−0.15	0.003*
		SC	T1	48.9	1.3	0.027	48.3	49.5	−0.15	−0.30	
			T2	55.2	1.1	0.020	54.6	55.8	−0.18	−0.28	0.004*
Team sports	F	CA	T1	33.3	1.0	0.030	32.7	33.9	0.20	−0.05	
			T2	28.5	1.1	0.039	27.9	29.1	0.12	−0.10	0.001*
		SA	T1	34.2	1.0	0.029	33.6	34.8	0.22	−0.08	
			T2	31.5	1.1	0.035	30.9	32.1	0.15	−0.12	0.002*
		SC	T1	47.6	1.4	0.029	47.0	48.2	−0.20	−0.28	
			T2	52.2	1.2	0.023	51.6	52.8	−0.22	−0.30	0.003*
	M	CA	T1	35.1	1.1	0.031	34.5	35.7	0.25	−0.04	
			T2	29.7	1.3	0.044	29.1	30.3	0.18	−0.10	0.002*
		SA	T1	36.0	1.2	0.033	35.4	36.6	0.27	−0.08	
			T2	33.3	1.2	0.036	32.7	33.9	0.20	−0.12	0.003*
		SC	T1	45.8	1.5	0.033	45.2	46.4	−0.25	−0.35	
			T2	50.8	1.4	0.028	50.2	51.4	−0.28	−0.38	0.004*

X, mean; SD, standard deviation; CV, coefficient of variation; CI95%, confidence interval; p, significance threshold at *p* < 0.05 marked with *; CA, cognitive anxiety; SA, somatic anxiety; SC, self-confidence.

Overall, for SMS-II, participants showed positive shifts in autonomous motivation (intrinsic, integrated, and identified regulation, from T1 to T2, particularly in groups that received the intervention). Across both genders and sport types, Intrinsic Motivation increased significantly, with the greatest gains observed in female athletes in individual sports (T1 M = 17.7, T2 M = 19.2). Similarly, Integrated Regulation and Identified Regulation exhibited statistically substantial improvements across all subgroups.

In contrast, the more controlled forms of motivation—Introjected and External Regulation—declined significantly post-intervention, reflecting a shift away from ego-driven or reward-based engagement. Amotivation also decreased notably, particularly among male athletes in team sports (T1 M = 8.2, T2 M = 6.8, *p* = 0.015), indicating an improvement in psychological engagement with sport. Descriptively, coefficients of variation remained low across all subscales (CV < 0.075), suggesting homogeneity within subgroups. Skewness and kurtosis values fell within acceptable ranges (−0.3 to +0.3), supporting the assumption of normal distribution for parametric analysis.

The CSAI-2 subscales revealed statistically and practically considerable gains in psychological readiness for competition. Cognitive Anxiety and Somatic Anxiety levels were consistently lower at T2 across all subgroups. For example, female team-sport athletes demonstrated a decrease in Cognitive Anxiety from T1 (*M* = 33.3) to T2 (*M* = 28.5, *p* = 0.001), while male individual-sport athletes showed reductions in Somatic Anxiety (T1 M = 32.1, T2 M = 29.7, *p* = 0.003).

Conversely, self-confidence scores demonstrated a pronounced rise in every subgroup. The most notable improvement was observed in female individual-sport athletes (T1 M = 50.8, T2 M = 56.7, *p* = 0.003), indicating that the psychological skills training was particularly effective in this population. As with SMS-II, CV values were acceptably low (<0.05 for most subscales), and the normality assumptions were upheld. Skewness and kurtosis values were minimal, further supporting the robustness of the descriptive statistics.

Paired-samples t-tests were conducted to assess changes in athletes’ motivation and anxiety levels following the 4-week psychological intervention. Statistically significant differences were observed across all SMS-II and CSAI-2 subscales between the pre-intervention (T1) and post-intervention (T2) assessments.

Substantial improvements were observed in all SMS-II subscales following the intervention. The effect sizes, as measured by partial eta squared (*η*^2^), were large for intrinsic motivation (*η*^2^ = 0.457), integrated regulation (*η*^2^ = 0.403), and identified regulation (*η*^2^ = 0.412), confirming substantial gains in autonomous motivation. Reductions in amotivation were also supported by a strong effect (*η*^2^ = 0.231). Similarly, CSAI-2 results revealed large effects for cognitive anxiety reduction (*η*^2^ = 0.514), somatic anxiety reduction (*η*^2^ = 0.357), and increased self-confidence (*η*^2^ = 0.485), all reflecting robust improvements in psychological readiness.

Across all subgroups, there was a consistent increase in autonomous forms of motivation, including Intrinsic Motivation (IM), Integrated Regulation (INR), and Identified Regulation (IDR). For example, individual-sport female athletes showed a mean increase of ΔX = 1.5 (ΔSD = 1.2) in IM [*t*(45) = 5.12, *p* = 0.003, *d* = 1.25], indicating a large effect. Similar patterns were observed in male and team-sport participants, with effect sizes ranging from *d* = 0.98 to *d* = 1.23, suggesting good improvements.

In contrast, controlled forms of motivation – Introjected Regulation (INTJ) and External Regulation (EXT) – showed significant reductions, with moderate effect sizes (EXT in male team-sport athletes: ΔX = −1.2, ΔSD = 1.1, *p* = 0.030, *d* = 0.61). Amotivation (AMOT) also declined across all groups, with the strongest reduction in individual-sport men (ΔX = −1.9, ΔSD = 1.5, *p* = 0.017, *d* = 0.90), indicating a clinically meaningful gain in sport engagement. Regarding competitive anxiety (CSAI-2), Cognitive Anxiety (CA), and Somatic Anxiety (SA) levels, they significantly decreased post-intervention across all groups. Female team-sport athletes demonstrated one of the largest changes in CA (ΔX = −4.8, ΔSD = 2.8, *p* = 0.001, *d* = 1.50), indicating a substantial decrease in pre-competition worry. Concurrently, Self-Confidence (SC) increased markedly in every subgroup, especially among individual-sport women (ΔX = 5.9, ΔSD = 3.3, *p* = 0.003, *d* = 1.49).

Effect sizes across subscales ranged from moderate (*d* ≈ 0.6) to very large (*d* > 1.4), reinforcing the efficacy of the psychological training program. These results collectively indicate that the intervention not only enhanced autonomous motivation and reduced anxiety but also significantly improved psychological readiness for competition, particularly among female athletes and those in individual sports (Table 5).

**Table 5:** Paired samples *t*-test T1 vs. T2.

Sport	Gender	Subscale	*X* (T1)	*X* (T2)	Δ*X*	ΔSD	*t*(df)	*p*	*d*
Individual sports	F	IM	17.7	19.2	1.5	1.2	5.12	0.003*	1.25
		INR	17.1	18.3	1.2	1.1	4.67	0.005*	1.13
		IDR	17.5	18.9	1.4	1.2	4.91	0.004*	1.20
		INTJ	12.6	11.1	−1.5	1.3	3.21	0.030*	0.78
		EXT	10.2	9.3	−0.9	1.1	2.87	0.022*	0.69
		AMOT	6.3	4.5	−1.8	1.4	3.89	0.010*	0.94
		CA	28.8	24.3	−4.5	2.9	6.22	0.001*	1.52
		SA	29.4	27.6	−1.8	1.6	4.01	0.002*	0.98
		SC	50.8	56.7	5.9	3.3	6.00	0.003*	1.49
	M	IM	16.8	18.6	1.8	1.3	5.06	0.005*	1.23
		INR	16.4	17.7	1.3	1.2	3.99	0.012*	0.97
		IDR	17.0	18.4	1.4	1.2	4.15	0.009*	1.01
		INTJ	13.3	11.7	−1.6	1.4	3.01	0.038*	0.73
		EXT	10.8	9.9	−0.9	1.1	2.79	0.031*	0.67
		AMOT	7.1	5.2	−1.9	1.5	3.71	0.017*	0.90
		CA	31.5	26.1	−5.4	3.0	6.02	0.002*	1.48
		SA	32.1	29.7	−2.4	2.1	3.92	0.003*	0.96
		SC	48.9	55.2	6.3	3.4	6.23	0.004*	1.53
Team sports	F	IM	16.2	18.0	1.8	1.3	4.77	0.004*	1.18
		INR	15.8	17.4	1.6	1.2	4.12	0.007*	1.00
		IDR	16.4	18.3	1.9	1.3	4.21	0.008*	1.03
		INTJ	14.1	12.3	−1.8	1.4	3.01	0.035*	0.73
		EXT	11.4	10.2	−1.2	1.2	2.56	0.026*	0.62
		AMOT	7.5	6.1	−1.4	1.3	3.11	0.012*	0.75
		CA	33.3	28.5	−4.8	2.8	6.11	0.001*	1.50
		SA	34.2	31.5	−2.7	2.2	3.90	0.002*	0.95
		SC	47.6	52.2	4.6	3.2	5.77	0.003*	1.44
	M	IM	15.6	17.4	1.8	1.3	4.89	0.006*	1.21
		INR	15.1	17.1	2.0	1.3	4.05	0.009*	0.98
		IDR	15.9	17.9	2.0	1.3	4.20	0.011*	1.02
		INTJ	14.7	12.9	−1.8	1.3	2.81	0.042*	0.68
		EXT	12.0	10.8	−1.2	1.1	2.50	0.030*	0.61
		AMOT	8.2	6.8	−1.4	1.2	2.99	0.015*	0.72
		CA	35.1	29.7	−5.4	3.0	6.05	0.002*	1.49
		SA	36.0	33.3	−2.7	2.1	3.88	0.003*	0.94
		SC	45.8	50.8	5.0	3.3	5.91	0.004*	1.48

ΔX, mean differences; ΔSD, standard deviation differences; *t*(df), *t*-statistic with degrees of freedom from a paired-samples *t*-test, *p < 0.05 statistical significance; *d*, Cohen’s *d* standardized effect size.

A series of 2 (Time: T1, T2) × 2 (Gender: women and men) × 2 (Sport Type: Individual, Team) mixed ANOVAs were conducted on each subscale of the SMS-II and CSAI-2 questionnaires to assess the effects of the 4-week psychological skills training program. Below, in Tables 6, 7, we report the main effects of Time (within-subjects), as well as the interaction effects with Gender and Sport Type (between-subjects).

**Table 6:** Mixed ANOVA results by SMS-II subscales.

Subscale	Effect	*F*	*p*	*p* Bonf.	Partial *η*^2^
IM	Time	97.56	0.001	0.009*	0.457
	Time × Gender	1.55	0.216	1.000	0.013
	Time × Sport type	6.33	0.014*	0.126	0.052
INR	Time	78.40	0.001	0.009*	0.403
	Time × Sport type	5.90	0.018*	0.162	0.048
IDR	Time	81.22	0.001	0.009*	0.412
	Time × Gender	2.30	0.131	1.000	0.019
INTJ	Time	21.10	0.001	0.009*	0.154
	Time × Sport type	3.87	0.051	0.459	0.032
EXT	Time	19.54	0.001	0.009*	0.144
	Time × Gender	2.70	0.102	0.918	0.023
AMOT	Time	34.87	0.001	0.009*	0.231
	Time × Sport type	4.12	0.045*	0.405	0.034

F, F-ratio from repeated-measures ANOVA; *p*, statistical significance threshold; Partial *η*^2^, partial eta squared effect size, *p* < 0.05 indicates statistical significance (marked with an asterisk); *p* Bonf., adjusted *p* after Bonferroni.

**Table 7:** Mixed ANOVA results by CSAI-2 subscales.

Subscale	Effect	*F*	*p*	*p* Bonf.	Partial *η*^2^
CA	Time	122.65	0.001	0.009*	0.514
	Time × Gender	1.89	0.173	1.000	0.016
	Time × Sport type	7.30	0.008*	0.072	0.059
SA	Time	64.45	0.001	0.009*	0.357
	Time × Gender	2.23	0.138	1.000	0.019
	Time × Sport type	3.99	0.048*	0.432	0.033
SC	Time	109.33	0.001	0.009*	0.485
	Time × Gender	2.75	0.100	0.900	0.023
	Time × Sport type	4.88	0.030*	0.270	0.004

F, F-ratio from repeated-measures ANOVA; *p*, statistical significance threshold; Partial *η*^2^, partial eta squared effect size, *p* < 0.05 indicates statistical significance (marked with an asterisk); *p* Bonf., adjusted *p* after Bonferroni.

Significant main effects of time were observed across all six SMS-II subscales, indicating that the intervention resulted in robust psychological changes in motivation patterns. Autonomous motivation exhibited a pronounced gain, with large effects in Intrinsic Motivation (IM) (*F* = 97.56, *p* < 0.001, *η*^2^ = 0.457), Integrated Regulation (INR), and Identified Regulation (IDR) (*η*^2^ > 0.44), showing that athletes became more self-determined post-intervention. Conversely, controlled motivation forms—Introjected Regulation (INTJ) and External Regulation (EXT)—showed significant decreases over time, although with moderate effect sizes. Amotivation (AMOT) significantly decreased (*F* = 34.87, *p* < 0.001, *η*^2^ = 0.231), suggesting enhanced psychological engagement in sport.

There were no significant Time × Gender interactions (*p* > 0.05), suggesting that male and female athletes benefited similarly from the intervention. However, Time × Sport Type interactions emerged in multiple subscales—including IM, INR, AMOT, and marginally INTJ—indicating that individual-sport athletes experienced greater motivational gains than team-sport athletes. These effects were particularly evident in the improvement of intrinsic and integrated motivation.

All three CSAI-2 subscales showed statistically significant main effects of time: Cognitive Anxiety (CA) and Somatic Anxiety (SA) both substantially reduced from T1 to T2 across all groups (*F* = 122.65 and 64.45, respectively; *p* < 0.001; *η*^2^ > 0.514), suggesting reduced pre-competition stress levels. Self-Confidence (SC) substantially enhances post-intervention (*F* = 109.33, *p* < 0.001, *η*^2^ = 0.485), representing a highly desirable psychological shift.

No significant time × gender effects were observed for anxiety or confidence, again suggesting a uniform benefit across male and female athletes. In contrast, Time × Sport Type interactions were statistically significant for all three CSAI-2 subscales (*p* < 0.05), indicating that individual-sport athletes experienced larger reductions in anxiety and greater increases in self-confidence compared to those in team sports.

To control for Type I error inflation due to multiple comparisons across subscales, a Bonferroni correction was applied, adjusting the significance threshold to *p* < 0.0056. Following this correction, only the main effects of time across all SMS-II and CSAI-2 subscales remained statistically significant. This indicates a robust and consistent effect of the psychological intervention on both sport motivation and anxiety regulation, irrespective of sport type or gender.

All Time × Gender and Time × Sport Type interaction effects did not retain significance after correction, suggesting that the intervention’s effects were generalizable across subgroups, rather than specific to any one gender or sport category.

## 4 Discussion

This study investigated the efficacy of a structured 4-week psychological intervention – comprising goal-setting workshops, visualization (Predoiu et al., 2020; Pelz, 2025), mindfulness, and team cohesion exercises (Reardon and Hitchcock, 2024; Michel, 2019; Appelbaum et al., 2020) – on athletes’ motivation and competitive readiness (Kaplánová, 2024; Beck et al., 2024). Using a sample of 512 participants across individual and team sports, we observed robust improvements in autonomous motivation (as measured by SMS-II), marked reductions in both cognitive and somatic anxiety (Kais and Raudsepp, 2004; Styck et al., 2022; Fox and Houston, 1983), and significant increases in self-confidence (CSAI-2).

The intervention’s impact on motivation and anxiety was not only demonstrated as a clear effect, but also practically meaningful, with large effect sizes across key variables. For instance, *η*^2^ = 0.457 for intrinsic motivation and *η*^2^ = 0.514 for cognitive anxiety reduction suggest that nearly half the variance in these outcomes was attributable to the intervention itself, reinforcing its empirical strength.

Our findings align with Self-Determination Theory (SDT), which emphasizes the importance of satisfying three basic psychological needs—autonomy, competence, and relatedness (Maddens et al., 2023; Zhang and Miao, 2024)—to foster intrinsic motivation and well-being. The significant increases in intrinsic motivation, integrated, and identified regulation, alongside reductions in controlled forms of motivation and amotivation (Tsorbatzoudis et al., 2006; Biese et al., 2024; Vlachopoulos, 2024), highlight how SDT-based strategies effectively transform motivational orientation in athletic populations. This finding is consistent with prior SDT-informed interventions, which have demonstrated their effectiveness in enhancing autonomous motivation and health-related outcomes.

The connection between the intervention outcomes and SDT can be further understood by examining the specific mechanisms through which each psychological skill supported basic psychological needs. Mindfulness training likely contributed to enhanced emotional regulation and present-moment awareness, satisfying the need for autonomy by allowing athletes to respond consciously rather than reactively to competitive stress. Goal-setting strategies played a crucial role in fostering a sense of competence by enabling athletes to set, pursue, and achieve self-endorsed objectives, thereby reinforcing their self-efficacy. Team cohesion exercises fostered feelings of belonging and mutual support, directly nurturing the need for relatedness. Together, these mechanisms may explain the observed increases in autonomous motivation and self-confidence, as well as reductions in anxiety. This alignment between intervention content and psychological need satisfaction reflects the theoretical coherence of the SDT framework and strengthens the interpretive validity of the results.

The intervention’s efficacy extended beyond motivation: Cognitive and somatic anxiety significantly decreased, while self-confidence surged. These findings reflect established sport psychology frameworks, demonstrating how psychological skills training enhances performance by regulating emotional arousal and bolstering confidence—key components of competitive readiness. The large effect sizes reported, even after Bonferroni correction, underscore the practical relevance of such interventions. However, given the consistently large effect sizes, it is important to interpret these results conservatively, as they may reflect measurement artifacts or context-specific influences in the absence of a control condition.

While intervention effects were statistically consistent across sport types and genders (Rodrigues et al., 2021; Omiya et al., 2014), a closer examination reveals nuanced differences that align with prior research. Individual-sport athletes demonstrated marginally greater gains in autonomous motivation and confidence, echoing findings that such athletes often show higher receptivity to sport psychology techniques and rely more on self-regulatory competencies rather than social dynamics (Jackman et al., 2024; Jordalen et al., 2016; Diotaiuti et al., 2021; O'Malley et al., 2024; Church et al., 2017). Conversely, team sports may foster psychological resilience through mechanisms such as social support, collective identity, and collaborative emotion regulation. Recent work indicates that while individual sports enhance self-efficacy, team sports uniquely promote social support, both pathways contributing to resilience, but via distinct processes (Wei et al., 2025). Although these sport-type differences were not statistically significant after correction, this framework can inform how interventions might be tailored – augmenting the social components for teams and enhancing autonomous strategies for individuals.

One possible explanation for the slightly greater gains observed in individual-sport athletes is that these mechanisms, such as self-monitoring and personal accountability for outcomes, play a role. Individual athletes often operate in performance contexts where success depends solely on their own preparation and execution, fostering greater self-regulatory behaviors. This heightened sense of personal responsibility may make them more receptive to interventions that enhance autonomy, goal orientation, and emotional regulation. In contrast, team athletes may benefit more from collective factors such as social support and shared responsibility, which might dilute the impact of individually focused psychological strategies.

Contrary to some past findings reporting higher anxiety levels in female athletes, our results showed gender-invariant intervention effects. This suggests that the intervention’s components—goal setting, mindfulness, and cohesion—resonate similarly across genders, reinforcing the value of gender-general psychological training programs in sport.

The findings strongly support Hypotheses 1 and 2 (Confirmatory). Athletes exhibited clear shifts toward autonomous forms of motivation, including intrinsic motivation, integrated regulation, and identified regulation, alongside a reduction in amotivation and controlled regulation. This aligns with Self-Determination Theory and highlights the effectiveness of brief, structured psychological interventions. Hypothesis 2 was also confirmed, as all groups reported lower anxiety and increased self-confidence, even after correction for multiple comparisons. Hypotheses 3 and 4 (Exploratory), predicting moderation by sport type and gender, received only partial support. While individual-sport athletes displayed slightly greater gains in motivation and confidence, these differences did not remain statistically robust after adjustment. Gender did not significantly influence outcomes, suggesting that the intervention was broadly effective across demographic groups.

### 4.1 Practical implications

Translating these findings into practice, coaches and sport psychologists can leverage compact, SDT-informed programs to enhance motivation and emotional readiness across varied athletic contexts. Incorporating modules that strengthen autonomy (goal setting), competence (mental rehearsal), and relatedness (team cohesion) ensures the holistic development of a motivation and performance mindset.

### 4.2 Limitations and future research

Several opportunities remain for deepening our understanding. First, longitudinal follow-up could clarify the durability of these psychological gains across competitive seasons. Second, qualitative inquiry might illuminate athletes’ subjective experiences – how goal setting or mindfulness translates into internal cognitive shifts. Third, the inclusion of mediators (e.g., resilience, emotional regulation) could help map the mechanisms underlying motivation-anxiety interactions. Finally, exploring the personalization of interventions—e.g., autonomy strategies for individuals versus relational emphasis for team athletes—may enhance efficacy.

Second, several potentially influential individual factors—such as athletes’ years of competitive experience, prior exposure to psychological skills training, or duration of participation in elite sport- were not controlled for in the present analysis. These variables could function as uncontrolled moderators that influence how athletes respond to psychological interventions. Future research should investigate how such individual differences may mediate or moderate the outcomes of interventions, enabling more personalized and effective psychological training approaches.

Finally, the study did not include longitudinal follow-up assessments, limiting our ability to determine the durability of psychological changes over time. Future studies should examine whether gains in motivation and anxiety reduction are sustained across multiple competitive seasons. The exclusive reliance on self-report instruments (SMS-II and CSAI-2) introduces potential bias related to social desirability and demand characteristics, particularly given that coaches implemented the intervention and reported fidelity.

Given the short duration of the intervention and the absence of long-term follow-up, the sustainability of these effects remains uncertain and should be addressed in future longitudinal research.

Although the large sample size ensured sufficient statistical power, it may also have contributed to the detection of statistically significant but potentially small or trivial effects. Moreover, the consistently large effect sizes across multiple outcomes should be interpreted cautiously. Intervention delivery by coaches, combined with self-reported fidelity checks, introduces the possibility that expectancy or demand characteristics may influence outcomes. Future studies would benefit from incorporating blinded delivery, independent fidelity assessments, and mixed-method approaches to confirm the robustness of these effects.

## 5 Conclusion

This study demonstrated that a structured 4-week psychological intervention significantly improved athletes’ motivation profiles, reduced competitive anxiety, and enhanced self-confidence across both individual and team sports. Grounded in self-determination theory, the intervention promoted a shift toward more autonomous forms of motivation while diminishing amotivation and external regulation, suggesting its strong capacity to enhance internal drive and readiness for performance.

Importantly, the intervention was equally effective across genders and sport types, supporting its generalizability and practical applicability. The statistically and clinically meaningful reductions in cognitive and somatic anxiety, accompanied by large gains in self-confidence, emphasize the relevance of psychological skills training in competitive contexts. The consistency of these effects across a large and diverse sample, combined with the high statistical power, lends strong support to the robustness and reliability of the findings.

Two of the four hypotheses (H1 and H2) were fully supported; one (H3) was partially supported, and one (H4) was not supported. These results reinforce the value of targeted psychological interventions in enhancing motivation and emotional psychological readiness for competition, while also highlighting areas for further exploration, such as personalized intervention design based on sport type or gender.

These results underscore the value of embedding brief, evidence-based psychological training modules—such as goal setting, visualization, mindfulness, and team cohesion—into athletes’ preparation routines. Coaches and sport psychologists are encouraged to adopt and adapt such interventions as part of an integrated approach to enhance athletes’ psychological readiness for competition. Future research should investigate the long-term sustainability of these outcomes, the mechanisms of change, and the potential for personalization based on factors such as sport, gender, or psychological profile.

## Data Availability

The raw data supporting the conclusions of this article will be made available by the authors, without undue reservation.
